# Draft genome sequence of bacterium *Bacillus proteolyticus* strain IMGN4 from soil

**DOI:** 10.1128/mra.00459-24

**Published:** 2024-07-05

**Authors:** Yeongjun Kim, Sangmin Lee, Eunjeong Kim, Jeong A. Han, Eun Yu Kim, Ho-Seok Lee

**Affiliations:** 1 Center for Genome Engineering, Institute for Basic Science, Daejeon, South Korea; 2 Department of Biology, College of Sciences, Kyung Hee University, Seoul, South Korea; 3 KNU G-LAMP Research Center, KNU Institute of Basic Sciences, BK21 FOUR KNU Creative BioResearch Group, Department of Biology, College of Natural Sciences, Kyungpook National University, Daegu, South Korea; 4 Gyeonggido Agricultural Research & Extension Services, Hwaseong, South Korea; 5 Division of Natural and Applied Sciences, Duke Kunshan University, Kunshan, Jiangsu, China; 6 Environment Research Center, Duke Kunshan University, Kunshan, Jiangsu, China; DOE Joint Genome Institute, Berkeley, California, USA

**Keywords:** *Bacillus proteolyticus*, protease activity, antibiotic activity, biocontrol agent

## Abstract

Here, we present the draft genome of *Bacillus proteolyticus* IMGN4, the gram-positive, soil-dwelling bacterium discovered in mountain Maemi, Republic of Korea in May 2019. The assembly resulted in 7 contigs, comprising a total of 6,063,502 base pairs and have 6,115 coding sequences.

## ANNOUNCEMENT

*Bacillus proteolyticus* is a gram-positive, soil-dwelling bacterium known for its extensive proteolytic activity, contributing to the natural decomposition process and antibiotic activity (1, 2). Like other members of the *Bacillus* genus, *Bacillus proteolyticus* exhibits antibiotic activities that inhibit the growth of pathogenic organisms, especially due to its protease activity (3, 4). This draft genome can help uncover the genetic foundations of its proteolytic mechanisms, thereby enriching our understanding of its agricultural potential and promoting advancements in bioremediation.

The organism was isolated from soil collected in (37.23589, 127.08998), mountain Maemi, Republic of Korea in May 2019. The organism was isolated from a soil sample and spread onto TSA plate. Incubation was performed at 37°C for 16 hours in dark conditions and streaked twice to obtain a pure colony.

Genomic DNA was extracted from a single colony using Maxwell RSC Tissue DNA kit protocol. The extracted genomic DNA was divided into two portions for different sequencing methods. First, one portion was sheared with the Megaruptor 3 (Diagenode) and purified using AMPure PB magnetic beads (Pacific Biosciences) for size selection. The PacBio sequencing library was prepared using the PacBio SMRTbell prep kit 3.0. Subsequently HiFi sequencing was performed on the PacBio Sequel II, yielding 111,305 reads. The mean length of HiFi reads was 8,732 base pairs, with a maximum length of 26,196 base pairs, a minimum length of 923 base pairs, and an N50 value of 9,333 base pairs.

Second, the other portion was used to prepare the Illumina sequencing library using the TruSeq DNA Nano Library Prep Kit (Illumina, Inc., San Diego, CA, USA). Subsequently sequencing was performed on the Illumina HiSeq X Ten platform using a 2 × 150 paired-end protocol, yielding total 15,599,920 reads. Additionally, quality filtering and adaptor trimming were performed using Trimmomatic v0.38 (5) ensuring a phred score of 30 or higher in 90% of the bases.

The HiFi reads were assembled *de novo* using HGAP v4 (6) to generate the draft genome assembly. Following the draft assembly, Illumina reads were used to polish the genome with Pilon v1.21 (7) three times. The final draft genome of *Bacillus proteolyticus* IMGN4 consisted of 7 contig, totaling 6,063,502 base pairs, with an average GC content of 35.3% and an N50 value of 5,162,090. To assess assembly quality, BUSCO v5.1.3 (8) analysis was performed and found it highly complete (99.19%).

Prokka v1.14.6 (9) was used to predict genes, which yielded 6,115 coding sequences, 109 tRNA genes, and 42 rRNA genes. Annotation was carried out using psiblast v2.7.1 (10) against the EggNOG DB v4.5 (11). The assembled draft genome was compared to the Genome Taxonomy Database (GTDB) via GTDB-Tk v2.3.2 (12) and assigned to *Bacillus proteolyticus*. In each step, default parameters were used except where otherwise noted.

Recent studies have highlighted the beneficial roles of *Bacillus proteolyticus*, including its antibacterial activity (13), probiotic properties (14), and promotion of seedling growth (15). These findings emphasize the potential applications of this genome report as a valuable resource for understanding the biological mechanisms at the genomic level.

## Data Availability

The annotated draft genome sequence has been deposited in the NCBI under the DDBJ/ENA/GenBank accession number JBBMKW000000000. The version described in this paper is version JBBMKW010000000. The BioProject database accession number is PRJNA1092556, and the BioSample database accession number is SAMN40626903. The Sequence Read Archive information is available under the accession number SRR28579662, SRR28579663.

## AFTER EPUB

*[This article was published on 5 July 2024 missing Eun Yu Kim's contributions. The contributions were corrected in the current version, posted on 15 July 2024.]*
